# New Remedies

**Published:** 1867-08

**Authors:** J. G. Westmoreland

**Affiliations:** Prof. of Materia Medica and Therapeutics in Atlanta Medical College


### ARTICLE II.

*New Remedies.* By J. G. Westmoreland, M. D., Prof. of Materia Medica and Therapeutics in Atlanta Medical College.

The science of Medicine, in its several departments, is progressive. While the truths discovered and practiced by the ancients should not be disregarded, the exposure of false theories and erroneous practical conclusions should be made whenever discovered. Many of the truths of Physiology, Therapeutics, Chemistry, etc., now well established, have been discovered within the last half century. During that time, opinions not a few, entertained by the Profession previously, have been exploded by the onward march of scientific medicine.

In the discovery of new remedies, and the manner of their application in the treatment of disease, progression in medicine is more particularly marked. Facts have been acknowledged, and conclusions arrived at heretofore, without sufficient tests to establish their truth; and hence the frequent explosions of theories, recognised by their enthusiastic propounders as perfection in science.

Experiments and tests upon which a principle in medicine is to be established, should be so extended, under varied circumstances, that no doubt of its correctness can be entertained. Statistics, to be reliable then, must not only include many cases of the same character, on which the test or experiment has been made, but must be reported only after the test has been made under all contingencies, which may be likely to impress diseases, or influence the action of remedies. From a want of attention in this all important particular, principles most erroneous, and systems of practice most injurious, have been inaugurated.

In crowded hospitals of densely populated cities, diseases are properly treated by means not at all applicable to the same affections in the country. Unless this fact be borne in mind, important remedies may be discarded, and treatment instituted and promulgated that will be found not only useless, but decidedly injurious at another time. On this account, pneumonia, properly treated by the use of depletives and sedatives in the rural districts of Georgia, may be more successfully managed, in a London Hospital by constantly sustaining the vital energies by cerebral stimulants. The same may be said of certain epidemic influences, requiring vital stimulants in connection with any treatment used.

In disease, as with medicinal agents, there is, in the profession, a disposition to generalize, making the same remedy or mode of treatment applicable to a great variety of diseases, and under all the varied circumstances in which they occur. This labor-saving proclivity is productive of much embarrassment to the progress of scientific investigation; and lays the foundation for one-ideal systems of medicine. New remedies, to be appliedin the treatment of disease, on general therapeutic principles, and new systems of practice founded on the ridiculous notion of identity of disease, differ widely from each other. Nor do we think he who throws himself back on the labor-saving doctrine of *vis medicatrix naturæ*, is less a stumbling block to the young investigator than other charlatans. Drugs have their medicinal properties, and diseases their peculiar pathological characters, both of which must be known in order to the successful practice of medicine.

It is found that in classifications of materia medica, made with reference to the medicinal properties, several agents are included in many of the classes, differing from each other in the degree of activity, promptness, etc. Several properties are possessed also by some drugs, such as astringent, nervous tonic, emetic, etc., which give it a place in these classes respectively. Relief to diseased organs is often effected indirectly by the action of a remedy upon a healthy part. These are points of study in the selection of remedies. In agents for a long time used, new properties are some times discovered, or their known properties made subservient in a manner not before known. Thus, new remedies are not necessarily newly discovered agents, nor even newly discovered properties in known agents.

It is a duty of the physician towards his patient, to seek the most prompt and efficient palliative or cure for disease. It is his duty toward the profession, to make known, by publication in some way, any discovery or improvement made.

The blockaded condition of the Southern States during the late war, with its many evils, brought one good to the medical profession, and perhaps to humanity. It forced physicians, by the scarcity of drugs, to seek new agents in the abundantly supplied fields and forests of their own "sunny South."

We are glad to know that the necessity worked a taste for investigations in therapeutics, which will result in valuable additions to materia medica. Its benefits are already being realized by the profession, in the reports of valuable unofficial medical plants being constantly published in this and other journals. Some valuable contributions of this kind have been made by Dr. Phares, of Mississippi, and others. Our experience with some of them confirms the reports made by the authors alluded to.

It is gratifying also to know that the "Medical Association of Georgia" is alive to the importance of such investigations. At its last session, a committee was appointed to collect information on the properties and uses of "unofficinal indigenous plants," and report the same at its next annual meeting in Augusta, on the 2d Wednesday in April next. Being a member of that committee, the writer would be pleased to have contributions on this subject sent to the Journal for publication, or by private letter, as may be most agreeable to the author.

